# The neuromatrix theory: A necessary step toward multidimensional understanding of pain by medical students

**DOI:** 10.23938/ASSN.1175

**Published:** 2026-08-18

**Authors:** Daniel Alexander Huayamis Huasupoma, Grace Connie Riquelme Murguía

**Affiliations:** Universidad Privada San Juan Bautista Facultad de Ciencias de la Salud Estudiantes de la Escuela Profesional de Medicina Humana Lima Perú

## Dear Editor,

After a thorough and critical reading of the article *Musculoskeletal Pain and Health-Related Quality of Life in Spanish University Students of Health Sciences*^1^, we would like to offer our perspective on the authors’ proposed theoretical framework.

The study explains the central sensitization hypothesis, which posits that altered processing contributes to the generalization of musculoskeletal pain^1^. While central sensitization is a contributing mechanism in pain, it does not accurately represent the primary cause. Although central sensitization contributes to the understanding of the development of chronic musculoskeletal pain, the neuromatrix theory offers a conceptual model that views the painful experience as arising from sensory factors.

Central sensitization explains how pain signals are amplified, but the neuromatrix theory not only explains psychosocial and cognitive factors; it also encompasses contextual, affective, and sensory factors^2^. Therefore, the neuromatrix theory agrees that the experience of pain cannot be explained solely by neurophysiological mechanisms but must consider biopsychosocial interactions^3^.

The experience of pain is generated by a globally distributed neural network in the brain where the following factors interact:

- Sensory inputs: Physical workload and poor ergonomics during clinical rotations.
- Cognitive-evaluative inputs: Stress related to academic performance and uncertainty of medical internships^4^.
- Affective inputs: The emotional impact of contact with human suffering and sleep deprivation.

We suggest that the study would be enriched by integrating the neuromatrix theory proposed by Ronald Melzack in order to reach conclusions that integrate contextual, cognitive, and affective dimensions^5^. Omitting this concept limits a comprehensive understanding, especially when considering the multidimensional nature of pain and its impact on student health.

As future health professionals, we cannot ignore that central pain processing is not just a response to physical load; it is also a complex structure in which the environment and the psyche shape biology. This is supported by findings that interpret pain as the result of neurobiological mechanisms, as well as cognitive, contextual, and affective factors, all of which interact.
